# Pediatric Malignant Arrhythmias Caused by Rare Homozygous Genetic Variants in *TRDN*: A Comprehensive Interpretation

**DOI:** 10.3389/fped.2020.601708

**Published:** 2021-02-22

**Authors:** Georgia Sarquella-Brugada, Anna Fernandez-Falgueras, Sergi Cesar, Elena Arbelo, Paloma Jordà, Ana García-Álvarez, Jose Carlos Cruzalegui, Erika Fernanda Merchan, Victoria Fiol, Josep Brugada, Ramon Brugada, Oscar Campuzano

**Affiliations:** ^1^Arrhythmias Unit, Hospital Sant Joan de Déu, University of Barcelona, Barcelona, Spain; ^2^Medical Science Department, School of Medicine, University of Girona, Girona, Spain; ^3^Cardiovascular Genetics Center, University of Girona-IDIBGI, Girona, Spain; ^4^Arrhythmias Unit, Hospital Clinic, University of Barcelona-IDIBAPS, Barcelona, Spain; ^5^Centro de Investigación Biomédica en Red. Enfermedades Cardiovasculares (CIBERCV), Madrid, Spain; ^6^Cardiology Service, Hospital Josep Trueta, University of Girona, Girona, Spain

**Keywords:** sudden cardiac death, arrhythmias, pediatric, genetics, triadin

## Abstract

**Aim:** To perform a comprehensive phenotype-genotype correlation of all rare variants in Triadin leading to malignant arrhythmias in pediatrics.

**Methods:** Triadin knockout syndrome is a rare entity reported in pediatric population. This syndrome is caused by rare variants in the *TRDN* gene. Malignant ventricular arrhythmias and sudden cardiac death can be a primary manifestation of disease. Although pharmacological measures are effective, some patients require an implantable defibrillator due to high risk of arrhythmogenic episodes.

**Main Results:** Fourteen rare genetic alterations in *TRDN* have been reported to date. All of these potentially pathogenic alterations are located in a specific area of *TRDN*, highlighting this hot spot as an arrhythmogenic gene region.

**Conclusions:** Early recognition and comprehensive interpretation of alterations in Triadin are crucial to adopt preventive measures and avoid malignant arrhythmogenic episodes in pediatric population.

## Introduction

**Figure 1:**
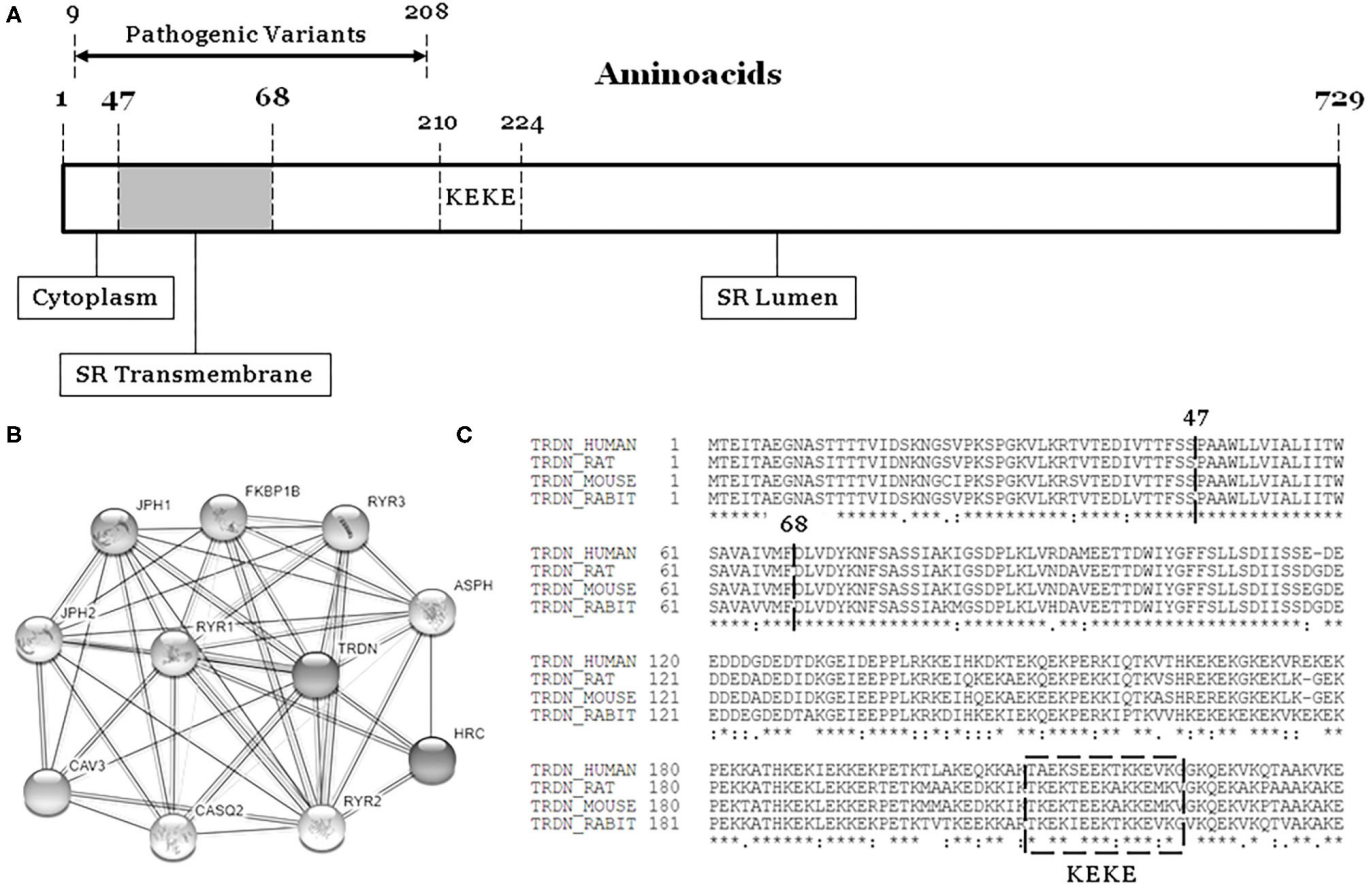
The TRDN structure and closest interaction network. **(A)** Three domains: Cytoplasm, Transmembrane, and Lumen. **(B)** Network of 10 closest proteins to Triadin. **(C)** Conservation between species of main regions containing pathogenic variants (9–208). SR, sarcoplasmatic reticulum.

The *TRDN* gene (HGNC: 12261, ID: 10345) encodes an integral transmembrane protein of the junctional sarcoplasmic reticulum called triadin, divided in cytoplasmatic, transmembrane and luminal domains (Figure 1) (1). Triadin forms a complex with ryanodine, junctin, and calsequestrin to create the sarcoplasmic reticulum calcium release unit. Therefore, triadin is essential for normal function of both cardiac and skeletal muscle, as reported in knock-out mouse models (2–4).

In 2012, a homozygous alteration in *TRDN* was reported in association with a malignant arrhythmogenic phenotype (5). Three years later, the term “Triadin KnockOut Syndrome (TKOS)” was proposed as a syndrome leading to high risk of arrhythmias caused by homozygous *TRDN* alterations, mainly in infants and young populations (6). However, a recent study demonstrated that TKOS is a rare clinical entity that does not contribute meaningfully to either sudden infant death syndrome or sudden unexplained death in the young (7).

The International TKOS Registry highlighted a correlation between rare *TRDN* variants in homozygotes and aggressive arrhythmogenic phenotypes characterized by T-wave inversion in precordial leads, transient QT prolongation, and recurrent ventricular arrhythmias. Although few genetic alterations in heterozygotes have been reported, they show moderate arrhythmogenic phenotypes (8). To date, a limited number of pathogenic alterations have been reported in *TRDN* (Figure 1, Table 1). All alterations considered pathogenic have been associated with recurrent episodes of ventricular fibrillation (VF), sudden cardiac arrest, and highly malignant forms of catecholaminergic polymorphic ventricular tachycardia (CPVT) or long QT syndrome (LQTS), particularly at early ages. However, patients do not show typical phenotypes of CPVT or LQTS, suggesting an overlapping arrhythmogenic phenotype that is highly lethal. We have performed a comprehensive analysis of all pathogenic and likely pathogenic variants reported thus far in *TRDN*.

**Table 1:** Genetic data of rare variants in *TRDN*.

**Nucleotide change**	**Protein change**	**Zygosity**	**dbSNP**	**EVS MAF (%) (EA/AA/All)**	**gnomAD (MAF%)**	**ClinVar (disease)**	**HGMD (disease)**	**ACMG score homozygosis**	**ACMG score heterozygosis**	**TRDN domain**
c.22+29A>G	IVS1dsA-G+29	Hetero	rs774068079	NA	1/247606 (0.0004%) Heterozygosis	NA	CS155261 (LQTS)	LP	VUS	C
c.53_56 delACAG	p.Asp18 Alafs^*^14	Hetero/ Homo	rs768049331	0.0/0.026/ 0.008 Heterozygosis	13/280480 (0.004%) Heterozygosis	P (CPVT)	CD124196 (CPVT)	P	VUS	C
Deletion Exon 2	-	Homo	NA	NA	NA	NA	CG1817756 (VF)	P	VUS	C/T
c.167T>C	p.Leu56Pro	Homo	NA	NA	NA	NA	NA	P	VUS	T
c.176C>G	p.Thr59Arg	Hetero	NA	NA	NA	NA	CM124195 (CPVT)	P	VUS	T
c.176C>T	p.Thr59Met	Hetero	rs397515459	NA	4/249154 (0.001%) Heterozygosis	VUS (CPVT)	CM193558 (CA)	LP	VUS	T
c.232+2T>A	IVS2dsT-A+2	Hetero	NA	NA	NA	NA	CS193557 (CA)	P	LP	L
c.423delA	p.Glu142 Lysfs^*^33	Homo	NA	0.218/0.122/ 0.188 Heterozygosis	NA	NA	CD193555 (CA)	P	LP	L
c.438_442 delTAAGA	p.Lys147^*^	Hetero/ Homo	rs970179891	NA	2/89152 (0.002%) Heterozygosis	NA	CD155260 (LQTS)	P	LB	L
c.484+1189G>A (c.485-24G>A)	-	Hetero	NA	NA	NA	NA	NA	P	P	L
c.502G>T	p.Glu168^*^	Hetero	NA	NA	NA	NA	CM160950 (CA)	P	LP	L
c.545dupA	p.Lys183Glufs^*^9	Homo	NA	NA	NA	NA	CI193559 (CA)	P	LP	L
c.613C>T	p.Gln205^*^	Hetero/ Homo	rs397515458	NA	5/163614 (0.003%) Heterozygosis	P (CPVT)	CM124194 (CPVT)	P	VUS	L
c.618delG	p.Ala208 Leufs^*^15	Homo	NA	NA	NA	NA	CD193556 (CA)	P	LP	L

*ACMG, American College of Medical Genetics and Genomics; C, cytoplasmatic; CA, cardiac arrest; ClinVar, clinical variation; CPVT, catecholaminergic polymorphic ventricular tachycardia; DM, disease mutation; EVS, exome variant server (EA, European-American; AA, African-American; All, all populations); gnomAD, genome aggregation database; HGMD, human genome mutation database; L, luminal; LB, likely benign; LQTS, long QT syndrome; LP, likely pathogenic; MAF, minor allele frequency; NA, no data available; P, pathogenic; T, transmembrane; VF, ventricular fibrillation; VUS, variant of uncertain significance*.

## Methods

We exhaustively reviewed the literature reporting *TRDN* and cardiac features up to August 2020. Data were collected from Human Genome Mutation Database (HGMD) (www.hgmd.org), ClinVar (www.ncbi.nlm.nih.gov/clinvar/intro), the National Center for Biotechnology Information SNP database (www.ncbi.nlm.nih.gov/SNP), Index Copernicus (https://www.indexcopernicus.com/index.php/en/), Google Scholar (scholar.google.es), Springer Link (link.springer.com), Science Direct (www.sciencedirect.com), Excerpta Medica Database (www.elsevier.com/solutions/embase-biomedical-research), and the IEEE Xplore Digital Library (ieeexplore.ieee.org/Xplore/home.jsp). Concerning *TRDN*, we consulted NCBI (https://www.ncbi.nlm.nih.gov/gene/?term=trdn), Genome Browser—Genomics Institute Sant Cruz, University of California (https://genome.ucsc.edu), GeneCards (https://www.genecards.org), and Genetics Home Reference (https://ghr.nlm.nih.gov). In addition, we obtained data for amino acid sequence or conservation among species (UniProt, www.uniprot.org) and protein–protein interactions (STRING, https://string-db.org).

Identified genetic variants were contrasted with variant data from Exome Variant Server (evs.gs.washington.edu/EVS) and Genome Aggregation Database (gnomad.broadinstitute.org, GnomAD), including recently added data concerning copy number variations. Genetic data were independently evaluated by three expert clinical geneticists and classified according to American College of Medical Genetics and Genomics (ACMG) guidelines (9). The PM2 item in the ACMG classification was considered fulfilled if minor allele frequency (MAF) in relevant population databases was ≤0.1% (10). For disease-causing variants, the majority of pathogenic variants were extremely rare in frequency (<0.001%) (11). PVS1 was only used for variants in genes with well-documented loss-of-function (www.ncbi.nlm.nih.gov/projects/dbvar/clingen) (12). Finally, all investigators discussed all data and agreed on final classification of all variants to avoid any bias.

## Results and Discussion

More than 20 years ago, triadin was stated as a key element maintaining regular heart rhythm via cardiac Ca^2+^ release, accompanied by its binding partners ryanodine-2, calsequestrin-2, and junctin (13, 14). However, the first association of alterations in *TRDN* as a cause of CPVT was not reported until 2012 (5). In this case, the authors reported two families showing a similar aggressive arrhythmogenic phenotype characterized by numerous polymorphic or bidirectional ventricular tachycardia. In the first family, from French West Indies, a homozygous deletion in exon 2 (c.del53_56, p.Asp18Alafs*14) was identified in a 2-year-old boy that presented with syncope followed by cardiac arrest at exercise. Resting electrocardiogram (ECG) following cardiac resuscitation showed numerous polymorphic or bidirectional ventricular extra beats and runs of polymorphic ventricular tachycardia. Our comprehensive analysis based on data currently available concluded a definite pathogenic role of this rare variant only in homozygous form (Table 1). In the second family, from Western France, two rare variants (c.176C>G, p.Thr59Arg/c.613C>T, p.Gln205*) were identified in a compound heterozygous form in a 26-year-old man with recurrent episodes of syncope during exercise since infancy. Exercise testing showed numerous bidirectional ventricular extra beats. Relatives carrying only one of these two variants did not show any clinical symptoms (5). Taking all data into account, we concluded that p.Thr59Arg may be highly deleterious in homozygous but not heterozygous form. In contrast, p.Gln205* seems to play a pathogenic role only in homozygosis (Table 1).

In 2015, Altmann et al. performed a comprehensive study in a cohort of 34 unrelated patients diagnosed with LQTS to identify the genetic cause of the disease (6). They identified five patients who showed similar aggressive arrhythmogenic phenotypes characterized by extensive T-wave inversion in precordial leads V1 through V4, with either persistent or transient QT prolongation or severe disease expression of exercise-induced cardiac arrest. Most patients were <10 years old and required aggressive therapy. Genetic analysis identified potential pathogenic rare *TRDN* variants in homozygous (c.del53_56/p.Asp18Alafs*14, and p.Lys147fs*) or compound heterozygous form (p.Lys147*, -c.438_442del-, and p.Asn9fs*5 -c.22+29A>G, IVS1dsA-G+29-). The p.Asp18Alafs*14 variant was identified in a young girl in homozygosis, and this pathogenic rare variant had already been identified by Roux-Buisson et al. (5). Three unrelated patients carried the same homozygous frameshift deletion (c.438_442del). Parents with the same rare variant in heterozygosis did not show any symptoms. Our comprehensive analysis based on currently available data concluded a definite pathogenic role of p.Lys147* but only in homozygous form (Table 1). The last patient was an infant boy carrying p.Lys147*/c.438_442del- and c.22+29A>G in a compound heterozygous form (6). Our comprehensive genetic analysis concluded that both p.Asn9fs*5 and p.Lys147* variants should remain classified as ambiguous significance in heterozygosis. Also, Rooryck et al. published in 2015 a family from Western France in which two young sisters suffered aggressive CPVT episodes (15). Both sisters carried c.613C>T/p.Glu205* and c.22+29A>G in heterozygosis. These two heterozygous rare variants were inherited from different parents, fitting with an autosomal recessive mode of inheritance. Both parents were asymptomatic. Our comprehensive genetic analysis concluded ambiguity of p.Asn9fs*5 and p.Gln205* in heterozygosis according to previous published data (Table 1). However, a combination of both rare variants seems to play a deleterious role.

One year later, Walsh et al. (16) reported two 2-year-old siblings who showed aggressive CPVT phenotypes with recurrent episodes of VF despite β-blockade and internal cardiac defibrillator implantation. A novel compound heterozygous pathogenic complex in *TRDN* was identified: p.Asp18Alafs*13, previously reported as pathogenic in the homozygous state, and c.502G>T/p.Glu168*, reported as novel. Each heterozygous variant was inherited from a different parent, both of whom remained asymptomatic. Our comprehensive genetic analysis concluded an ambiguous role of p.Asp18Alafs*13 in the heterozygous state and a potential pathogenic role of p.Glu168* both in homozygous and heterozygous states (Table 1).

In 2018, the first and only homozygous deletion of *TRDN* (exon 2) was published (17). The patient was a 16-month-old infant who presented the most severe arrhythmogenic phenotype described thus far—it was characterized by recovered cardiac arrest, recurrent VF despite beta-blockade and flecainide, T-wave inversion in anterior precordial leads, and prolonged rate-corrected QT of 490 ms. Neither parent ever showed arrhythmogenic symptoms, and genetic analysis identified the same deletion in both parents but in heterozygosis. In addition, the index case also carried a missense variant in *KCNE2* (c.170T>C, p. Ile57Thr), which was previously described in LQTS but is currently classified as likely benign mainly due to high population frequency (MAF: 0.105%). Our comprehensive genetic analysis concluded a pathogenic role of homozygous deletion in exon 2 of *TRDN* (Table 1).

In 2019, the first International TKOS Registry was launched (8). Data from its initial 21 patients showed that TKOS is a potentially lethal syndrome, mainly at a young age, and is characterized by T-wave inversions in precordial leads, transient QT prolongation, and recurrent VF despite pharmacological treatment. Five new rare and potentially pathogenic variants were identified: three in homozygosis (c.423del/p.Glu142Lysfs*33, c.545dup/p.Lys183Glufs*9, and c.618del/p.Ala208Leufs*15), and two in compound heterozygosis (c.232+2T>A/IVS2dsT-A+2, and c.176C>T/p.Thr59Met). Our comprehensive genetic analysis concluded a pathogenic role of all these rare variants but only in homozygous form (Table 1). Both variants in heterozygosis were classified as likely pathogenic (IVS2dsT-A+2) or ambiguous significance (p.Thr59Met) alone, but a combination of both in heterozygosis should be considered deleterious. Further, a novel homozygous rare variant (c.167T>C, p.Leu56Pro) in *TRDN* has recently been reported (18). This 2-year old boy was resuscitated from sudden cardiac arrest and had frequent VF episodes despite beta-blocker plus flecainide therapy. He received an implantable cardiac defibrillator (ICD). Both parents remained asymptomatic and carried the same rare variant but in heterozygosis. Functional studies reported in the same study support a pathogenic role for homozygous p.Leu56Pro. We definitely classify this rare variant in homozygosis as pathogenic (Table 1).

Recently, genome sequencing and *TRDN*-specific trio analysis were performed on a family (19). The index case was a 13-year-old boy who had his first cardiac arrest at the age of 18 months. He underwent placement of an ICD as well as left cardiac sympathetic denervation. Genetic analysis identified a maternally inherited c.22+29A>G variant, previously reported in 5 patients with TKOS from 3 unrelated families, leading to alternative splicing in the heterozygous form (6, 15). In addition, a novel deep intronic variant c.484+1189G>A (also annotated as c.485-24G>A) was identified in the index case. Functional studies determined that the last intronic variant not only disrupts proper splicing of exon 6a but also completely abolishes the CT1 transcript, ultimately leading to a *TRDN* null allele in the patient. Both parents had normal ECGs and negative personal and family histories of cardiac-related events. Taking all data into account, the variant c.22+29A>G in heterozygosis should be considered to have no conclusive deleterious role, while c.484+1189G>A should be classified as pathogenic (Table 1).

## Conclusions

Our retrospective study concludes that homozygous/compound heterozygous rare variants in *TRDN* are associated with highly malignant arrhythmogenic phenotypes. Arrhythmias usually occur at young ages, and pharmacological treatment is mandatory. Aborted sudden cardiac arrest is not rare, and implantable cardiac defibrillator is recommended to prevent new episodes. Pathogenic alterations are located in the first 208 amino acids of the protein, suggesting a hot spot associated with aggressive arrhythmogenic syndromes. Early identification and comprehensive analysis of rare *TRDN* variants may help adopt preventive measures to reduce risk of lethal episodes.

## Author Contributions

OC, EA, JB, and RB developed the concept. OC, AF-F, SC, PJ, AG-Á, JC, EM, and GS-B acquired, pre-processed, and analyzed the data. OC, AF-F, SC, VF, and GS-B prepared the manuscript. OC and RB supervised the study. All authors contributed to manuscript revision, read and approved the current submitted version.

## Conflict of Interest

The authors declare that the research was conducted in the absence of any commercial or financial relationships that could be construed as a potential conflict of interest.
